# Long-Term Stored Hemoglobin-Vesicles, a Cellular Type of Hemoglobin-Based Oxygen Carrier, Has Resuscitative Effects Comparable to That for Fresh Red Blood Cells in a Rat Model with Massive Hemorrhage without Post-Transfusion Lung Injury

**DOI:** 10.1371/journal.pone.0165557

**Published:** 2016-10-31

**Authors:** Masahiro Tokuno, Kazuaki Taguchi, Keishi Yamasaki, Hiromi Sakai, Masaki Otagiri

**Affiliations:** 1 Faculty of Pharmaceutical Sciences, Sojo University, Kumamoto, Japan; 2 DDS Research Institute, Sojo University, Kumamoto, Japan; 3 Department of Chemistry, Nara Medical University, Nara, Japan; Albany Medical College, UNITED STATES

## Abstract

Hemoglobin-vesicles (HbV), encapsulating highly concentrated human hemoglobin in liposomes, were developed as a substitute for red blood cells (RBC) and their safety and efficacy in transfusion therapy has been confirmed in previous studies. Although HbV suspensions are structurally and physicochemically stabile for least 1-year at room temperature, based on *in vitro* experiments, the issue of whether the use of long-term stored HbV after a massive hemorrhage can be effective in resuscitations without adverse, post-transfusion effects remains to be clarified. We report herein on a comparison of the systemic response and the induction of organ injuries in hemorrhagic shock model rats resuscitated using 1-year-stored HbV, freshly packed RBC (PRBC-0) and by 28-day-stored packed RBC (PRBC-28). The six-hour mortality after resuscitation was not significantly different among the groups. Arterial blood pressure and blood gas parameters revealed that, using HbV, recovery from the shock state was comparable to that when PRBC-0 was used. Although no significant change was observed in serum parameters reflecting liver and kidney injuries at 6 hours after resuscitation among the three resuscitation groups, results based on Evans Blue and protein leakage in bronchoalveolar lavage fluid, the lung wet/dry weight ratio and histopathological findings indicated that HbV as well as PRBC-0 was less predisposed to result in a post-transfusion lung injury than PRBC-28, as evidenced by low levels of myeloperoxidase accumulation and subsequent oxidative damage in the lung. The findings reported herein indicate that 1-year-stored HbV can effectively function as a resuscitative fluid without the induction of post-transfused lung injury and that it is comparable to fresh PRBC, suggesting that HbV is a promising RBC substitute with a long shelf-life.

## Introduction

Because red blood cell (RBC) transfusions are routinely used in clinical situations, they have greatly contributed to human health and welfare. Although there is little doubt that RBC transfusions are life-saving in most types of trauma that involve bleeding, it is impossible to completely eliminate some of the potential risks associated with conventional RBC transfusions, such as blood-type mismatching, infections by unrecognized pathogens, hepatitis or human immunodeficiency virus infections, etc. [1]. In addition, various complications that can arise after a RBC transfusion, such as transfusion-related acute lung injury (TRALI) and transfusion associated circulatory overload (TACO), have been reported in cases of patients with massive hemorrhages [1]. Furthermore, RBC products can undergo various biological changes during storage, including the accumulation of certain types of bioactive substances, loss of the deformability, decreasing oxygen (O_2_) delivery capacity, which are generally known as storage lesions [2]. This represents one of the reasons for why conventional RBCs are not permitted to be stored for 21–49 days in the world, depending on the country due to differences in additive solutions, storage conditions and method used to collect blood among countries (ex. 21 days in Japan, 42 days in USA) [3]. Therefore, it would be desirable to develop strategies for RBC transfusions or RBC transfusion alternatives that would enable them to be used safely and effectively after being stored for longer periods of time.

Hemoglobin-vesicles (HbV), a type of cellular type hemoglobin (Hb) based O_2_ carrier, were developed as a RBC substitute. In this product, a concentrated human Hb solution is encapsulated in a liposome, the surface of which is covered with polyethylene glycol (PEG) [4]. The transport capacity of O_2_ by HbV is equivalent to that for RBC, and HbV shows resuscitative effects that are comparable to RBC in hemorrhagic shock rat models [5–7]. In addition, HbV has been shown to be safe and biocompatible based on *in vitro* and *in vivo* experiments as follows: the absence of viral contamination [8], high biocompatibility in systemic immune responses [9], no innate toxicity [10–12], readily metabolized and excreted under both healthy and pathological conditions [13–15]. Furthermore, the structure and physicochemical characteristics of HbV in the liquid state remain about the same during storage for periods of over 1 year at 4, 23, and 40°C, indicating that a HbV suspension can be stored at room temperature for at least 1 year [16]. Based on these facts, HbV appears to have potential for use as a substitute for RBC and has the added benefit that it can be stored for much longer periods of time. However, it is not known whether resuscitation using long-term stored HbV after massive hemorrhage could affect the outcome *in vivo*, including resuscitative effects and transfusion-related adverse effects, compared to that for conventional RBC.

To evaluate whether long-term stored HbV effectively functions as an RBC substitute without inducing adverse, transfusion-related effects *in vivo*, we first compared the systemic response after the administration of 1-year-stored HbV and fresh packed RBC (PRBC-0) in a hemorrhagic shock model rat. In addition to 1-year-stored HbV and PRBC-0, we also prepared a 28-day-stored packed RBC preparation (PRBC-28) as a model of excessively stored PRBC and observed the systemic response as well. In subsequent experiments, we examined the induction of adverse, transfusion-related effects, with a focus on multi organ failure including hepatic, renal and lung injuries, after resuscitation by the above three preparations, and further investigated the reason for why HbV prevented the induction of post-transfused lung injury.

## Materials and Methods

### Animals and ethics statement

Male Lewis rats aged 10–12 weeks were purchased from Japan SLC, Inc (Shizuoka, Japan). The rats were housed in environmentally-controlled rooms with 12-hour light-dark cycles and allowed free access to chow and water. All animal experiments were reviewed and approved by the Animal Care and Use Committee of Sojo University (Permit number: 2015-P-026). The care and handling of the animals were in accordance with the NIH guidelines. In order to minimize any suffering of the animals, anesthesia (isoflurane) was used in surgical experiments with the depth of anesthesia being monitored and fluid administration was performed via a cannula without restraint. The rats were observed every 30 min throughout experiments and humanely sacrificed by an overdose of isoflurane when they met the following humane endpoint criteria after starting resuscitation: prostration, spasm, difficulty breathing, rotational motion and severe drop in blood pressure.

### Preparation of PRBC

Both PRBC-0 and PRBC-28 were prepared as described in a previous report with minor modifications [17]. In short, a cannula was introduced in Lewis rats (*n* = 32) *via* the carotid artery under isoflurane anesthesia. Whole blood (8–10 mL) was withdrawn in a syringe containing a CPDA-1 solution (Terumo Corporation, Tokyo, Japan), and the final concentration of the CPDA-1 solution was adjusted to 14%. Collected blood was passed through a high efficiency leukocyte reduction filter (Purecell^®^, Pall Corporation, NY, USA), and was subsequently centrifuged (1,000 g, 30 min) to separate the RBC from plasma. The final hematocrit (Hct) was adjusted to approximately 66% by removing a part of plasma phase and the preparation was then transferred to blood bags (Kawasumi Laboratories, Inc., Tokyo, Japan). PRBCs were stored at 4°C until used. The Hct value of PRBC-0 and PRBC-28 was 66.3 ± 0.5% and 61.4 ± 2.6%, respectively when used.

### Preparation of HbV

HbV particles were prepared under sterile conditions, as previously reported [18]. Briefly, an Hb solution was purified from outdated blood donated by the Japanese Red Cross Society (Tokyo, Japan). The encapsulated Hb (38 g/dl) contained 5.9 mM of pyridoxal 5'-phosphate as an allosteric effector for regulating O_2_ affinity. The lipid bilayer was a mixture of 1,2-dipalmitoyl-*sn*-glycero-3-phosphatidylcholine, cholesterol, and 1,5-bis-*O*-hexadecyl-*N*-succinyl-L-glutamate at a molar ratio of 5/4/0.9, and 1,2-distearoyl-*sn*-glycero-3-phosphatidyl-ethanolamine-*N*-PEG (0.3 mol%). The HbV particles were suspended in a physiological salt solution at [Hb] = 10 g/dL and [lipids] = 8 g/dL. The resulting suspension was deoxygenated by exposure to nitrogen gas prior to storage. The resulting HbV suspension was stored for 1 year at 4°C.

### Traumatic hemorrhagic shock and resuscitation

Traumatic hemorrhagic shock model rats were prepared as described in a previous report with minor modifications [17]. Lewis rats (*n* = 50) were anesthetized with isoflurane and a polyethylene catheter (PE-50 tubing, Becton Dickinson and Company, Tokyo, Japan) filled with heparinized saline (20 IU/mL) was placed in the right femoral artery for monitoring blood pressure and withdrawing blood, the femoral vein for infusing resuscitation fluids. Mean arterial pressure (MAP) and heart rate were monitored by means of an artery catheter connected to a pressure transducer coupled to a polygraph system (AD Instruments Inc., Nagoya, Japan). At about 16 hours after catheter placement, the anesthetized rats were subjected to a 5-cm midline laparotomy to induce trauma. After recovering from the anesthesia, hemorrhagic shock was induced by withdrawing blood via the arterial catheter into a heparinized syringe over a period of 10 minutes using a syringe pump (KD Scientific Inc., MA, USA) under ananesthesia. A volume comprised of 40% of the total blood volume, as estimated as 56 mL/kg body, was drawn [6]. The rats were maintained at MAP within 40 mmHg for 60 minutes by further blood withdrawal or a blood infusion. The total withdrawn blood volume was 24.1±1.4, 25.8±2.7 and 26.1±1.8 mL/kg for PRBC-0, PRBC-28 and HbV group, respectively. The resulting hemorrhagic shock rats were then infused with Ringer’s Lactate (RL) for over 40 minutes (Infused volume: twice the total of shed blood volume), and subsequently transfused with PRBC-0 (*n* = 12), PRBC-28 (*n* = 13) or HbV (*n* = 11) for over 30 minutes. The injected volume of PRBC was calculated as (Hct at the baseline)/(Hct in PRBC) × (bleeding volume), while that of HbV is calculated as [Hb at the baseline]/[Hb in HbV] × (bleeding volume). Hct and Hb concentrations were determined using an animal blood cell measuring apparatus (MEK-6458; NIHON KOHDEN, Tokyo, Japan). The infused volume and Hct of the PRBC and HbV were adjusted to individual values for the withdrawn blood by the addition of saline, resulting in nearly the same Hb dose among groups. No plasma or platelets were included in any of the resuscitative fluids used in this study. The sham treated rats (*n* = 10) were treated in an identical manner, except for bleeding and the infusion of a resuscitation fluid. Five rats of each group were randomly selected and injected with Evans Blue (EBD) (30 mg/kg, i.v.) after RL administration in order to evaluate EBD leakage and protein accumulation in bronchoalveolar lavage fluid (BALF). The remaining rats (*n* = 5) were used for the evaluation of blood gas, biological tests and histological tests. All surviving rats were followed up for up to 6 hours after the end of the transfusion. The following experiments were performed using rats euthanized by isoflurane overdose. All plasma samples were stored at -80°C for further use.

### Blood gas analysis, glucose concentration and Hct

Arterial blood samples were collected before hemorrhagic shock (baseline), immediately after hemorrhagic shock and resuscitation (end of transfusion) and at 6 hours after resuscitation. Blood gas parameters and lactate concentrations were determined using a iSTAT analyzer (Fuso Pharmaceutical Industries, Osaka, Japan). Hct was determined using an animal blood cell measuring apparatus (MEK-6458). Glucose concentrations were measured using commercial kits following the manufacturer’s instructions (Wako Pure Chemical Industries, Osaka, Japan).

### Blood biochemical tests

Collected blood samples (baseline and 6 hours after the end of the transfusion of each fluid) were centrifuged to obtain plasma. The levels of aspartate aminotransferase (AST), alanine aminotransferase (ALT), serum creatinine (CRE) and blood urea nitrogen (BUN) were determined using commercial kits following the manufacturer’s instructions (Wako Pure Chemical Industries, Osaka, Japan).

### Pulmonary edema evaluation

Pulmonary edema evaluations were performed according to a previous report with minor modifications [19]. Blood samples were collected at 6 hours after the EBD administration. A bronchoalveolar lavage was performed with 10 mL of saline, and the collected blood and BALF were centrifuged. The concentration of EBD in the plasma and BALF were determined based on the absorbance at 620 nm and are expressed as the percent of EBD that leaked into the BALF compared with the plasma EBD concentration. The percent of EBD leakage into the BALF was calculated as [EBD concentration in BALF]/[EBD concentration in plasma (6 hour after transfusion)] × 100. Protein concentration in BALF was also measured by means of a BCA protein assay kit (Thermo Scientific, IL, USA) for evaluation of protein accumulation. The lung wet to dry weight ratio was measured as another index of pulmonary edema [20]. At 6 hour after the administration of each fluid, left lung samples were removed and dried at 80°C for 48 hours, and the wet weight / dry weight ratio calculated.

### Histology

At 6 hour after the administration of each resuscitative fluid, the lobe of the right lung was excised and fixed in a neutral buffer solution containing 10% formalin, embedded in paraffin, and cut into sections. After removing the paraffin, the lung was stained with hematoxylin and eosin (HE) for histopathological evaluation. After the staining, the sections were observed using a BZ-X710 microscope (Keyence, Osaka, Japan). Histopathological scoring for lung tissue was performed as previously described with minor modifications. Thirty areas of lung sections were graded on a scale of 0–4 according to four parameters (congestion, edema, hemorrhage, and inflammation (cellular infiltration)) by pathologists and the data are expressed as the sum of the individual scores of these parameters [21]. For immunological staining, lung sections were treated with antigen retrieval in HistoVT one (Nacalai tesque Inc., Kyoto, Japan), solubilized in 50 mM Tris-HCl buffer containing 0.1% polysorbate 20 and blocked with Block Ace (Dainippon Pharmaceutics, Osaka, Japan). The lung sections were reacted overnight at 4°C with a diluted primary antibody, which contained the rabbit anti myeloperoxidase (MPO) heavy chain antibody (Santa Cruz, California, USA, cat#; SC-16128-R), or the mouse anti 8-hydroxy-2’-deoxyguanosine (8-OH-dG) antibody (Santa Cruz, California, USA, cat#; SC-66036). The secondary antibodies, Alexa Fluor 546 goat anti-rabbit IgG (Life technologies, Oregon, USA, cat#; A11010) for MPO or Alexa Fluor 488 goat anti-mouse IgG (Life technologies, Oregon, USA, cat#; A11001) for 8-OH-dG, were used as a molecular probe. The sections for MPO staining were counterstained with the 4',6-Diamidino-2-phenylindole (DAPI) solution (Dojindo Laboratories, Kumamoto, Japan, cat#; 340–07971). After the reaction, the slide was observed using a BZ-X710 microscope.

### Data analysis

Data are reported as the mean ± SD for the indicated number of animals. One-way repeated measures ANOVA with the Bonferroni collection was used for multiple comparisons within the same group. Differences compared among the groups at the same point in time were determined by ANOVA followed by Scheffe's method. Comparisons of values between before hemorrhage and at 6 hour after resuscitation in the same group were performed by paired t tests. Kruskal-Wallis test was performed for the histopathological evaluation. For survival studies, log-rank test with Bonferroni correction was used. Differences were considered to be significant when the value of *p* < 0.05.

## Results and Discussion

### Survival rate

**Fig 1:**
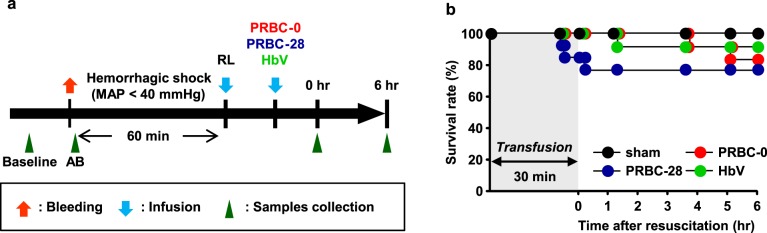
Scheme showing the experimental procedure and survival observations made after hemorrhagic shock and resuscitation with PRBC-0, PRBC-28 and HbV. (a) Scheme showing the development of hemorrhagic shock and resuscitation. (b) Survival rates of sham treated rats (*n* = 10), hemorrhagic shock rats resuscitated with PRBC-0 (*n* = 12), PRBC-28 (*n* = 13) or HbV (*n* = 11). AB indicates after bleeding. RL indicates Ringer’s Lactate.

Hemorrhagic shock was induced in the Lewis rats by withdrawing of 40% of their whole blood from the femoral artery, and were then maintained in a hypotensive state (MAP < 40 mmHg) for 60 min (Fig 1A). Four out of the forty (10%) hemorrhagic shock-induced rats died before each sample administration. The 6-hour survival rate in the PRBC-0 group was 83.3%, while that in the PRBC-28 group was slightly less (76.9%) (Fig 1B). This result is consistent with observations in humans, in which the stored RBC is associated with a risk of a poorer clinical outcome [22]. A higher 6-hour survival rate was found in the HbV group (90.9%). However, no significant difference was found among the groups.

### Systemic responses

**Fig 2:**
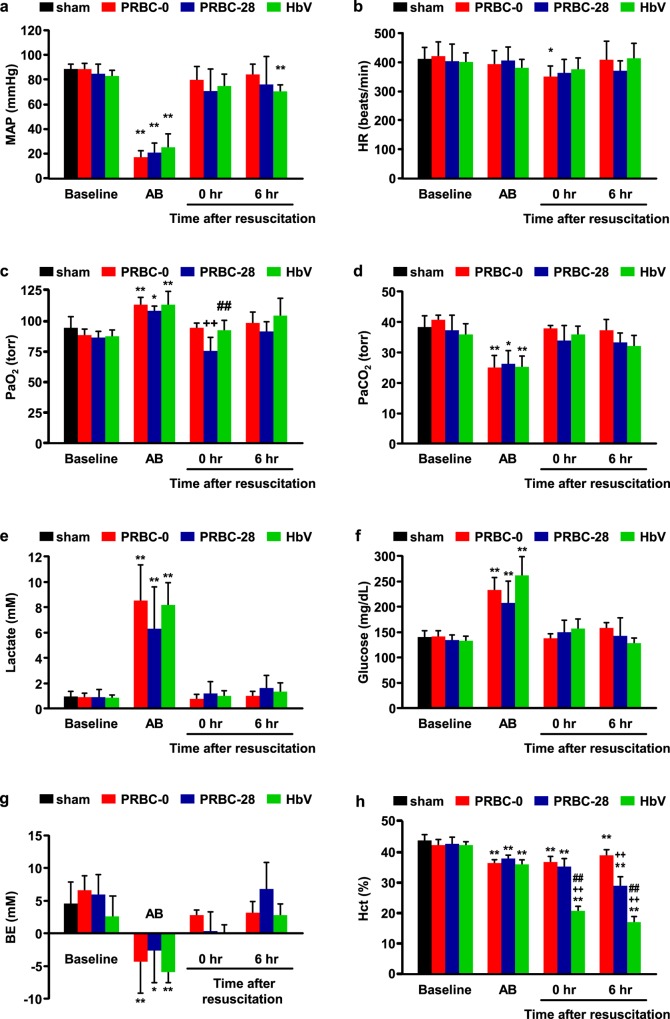
Changes in hemodynamic and blood gas parameters during and after resuscitation with PRBC-0, PRBC-28 and HbV. (a) Mean arterial pressure (MAP), (b) heart rate (HR), (c) arterial blood oxygen tension (PaO_2_), (d) arterial blood carbon dioxide tension (PaCO_2_), (e) lactate, (f) glucose, (g) base excess (BE) and (h) hematocrit (Hct) during and after hemorrhagic shock and resuscitation with PRBC-0, PRBC-28 or HbV for 6 hours. (a and b) Values are the mean ± SD; *n* = 10 each group. (c-g) Values are means ± SD; *n* = 5 each group. *p < 0.05 and **p < 0.01 vs. baseline, ^++^*p* < 0.01 vs. PRBC-0 group, ^#^*p* < 0.05 and ^##^*p* < 0.01 vs. PRBC-28 group. Rats that died during the observation period were excluded from the number. AB indicates after bleeding.

As shown in Fig 2A, MAP was decreased to below 40 mmHg immediately after hemorrhage. In both the PRBC-0 and PRBC-28 groups, it recovered to the level of the baseline at 0 hour after the administration of each sample and remained stable until planned death occurred (6 hour after resuscitation). On the other hand, the MAP after HbV administration was slightly decreased compared to the baseline. The reason for this is unclear, but circulatory blood flow in the HbV group might have been less than that in both PRBC groups because the HbV is captured by the mononuclear phagocyte system, mainly Kupffer cells and splenic macrophages [12,14]. In addition, the heart rate was slightly reduced after the end of administration of all of the resuscitative fluids (Fig 2B).

Arterial blood carbon dioxide tension (PaCO_2_) decreased significantly after bleeding, but completely recovered to the baseline level after the administration of the resuscitative fluids (Fig 2D). The values for arterial blood oxygen tension (PaO_2_) were significantly increased after the induction of hemorrhagic shock. After the administration of PRBC-0 and HbV, the PaO_2_ value reverted to the baseline level. However, the PaO_2_ value in the PRBC-28 group was lower than that for the other groups (Fig 2C).The values for glucose, lactate and base excess (BE) which reflect shock status recovered to the baseline level and were maintained there for 6 hours among all groups (Fig 2E–2G). In addition, the Hct values in all groups were reduced after bleeding (Fig 2H). After the administration of HbV, the Hct values were significantly reduced due to the dilution of the blood with the HbV solutions (HbV is not reflected to Hct values). Interestingly, the value of Hct at 6 hour after PRBC-28 administration decreased compared to that after PRBC-0 administration. These results indicate that all of the resuscitative fluids used in this study had the potential to resuscitate the rats from a hemorrhagic shock state.

### Hepatic injury

**Fig 3:**
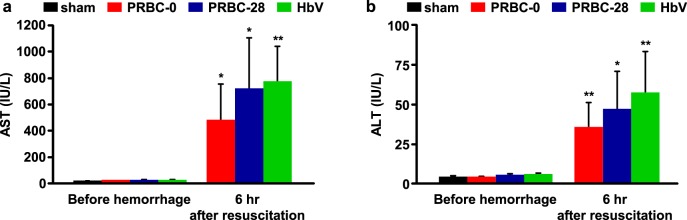
Evaluation of hepatic injury after hemorrhagic shock and resuscitation with PRBC-0, PRBC-28 and HbV. (a) Aspartate aminotransferase (AST) and (b) alanine aminotransferase (ALT) concentrations before bleeding and at 6 hours after resuscitation with PRBC-0, PRBC-28 or HbV. Values are the mean ± SD; *n* = 5 each group. *p < 0.05 and **p < 0.01 vs. before hemorrhage.

The levels of AST and ALT in plasma, which reflect hepatic injury, were significantly increased as the result of hemorrhagic shock and resuscitation for all samples (Fig 3A and 3B). It is known that systemic ischemia–reperfusion that have the potential to cause damage to the liver can be induced by RBC resuscitation from a massive hemorrhage [23]. Therefore, in this study, it would be induced by a hepatic ischemia–reperfusion injury caused by a massive hemorrhage and RBC or HbV resuscitation. Previous reports using a different hemorrhagic shock model showed that RBC or HbV resuscitation from hemorrhagic shock induced hepatic injury but returned to normal levels within a day [6,24]. These findings indicate that all resuscitative fluids used in this study would temporarily induce a mild hepatic injury due to ischemia–reperfusion.

### Renal injury

**Fig 4:**
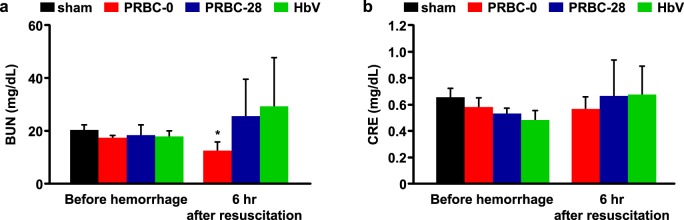
Evaluation of renal injury after hemorrhagic shock and resuscitation with PRBC-0, PRBC-28 and HbV. (a) Blood urea nitrogen (BUN) and (b) creatinine (CRE) levels before bleeding and at 6 hours after resuscitation with PRBC-0, PRBC-28 or HbV. Values are means ± SD; *n* = 5 each group. *p < 0.05 vs. before hemorrhage.

The CRE and BUN levels, which reflect renal function, showed some slight changes at 6 hour after the administration of resuscitative fluids (Fig 4A and 4B). Nevertheless, these values were not in abnormal ranges. Therefore, all of the resuscitative fluids used in this study do not appear to have had a meaningful functional effect on pathological changes in the kidney.

### Lung injury

**Fig 5:**
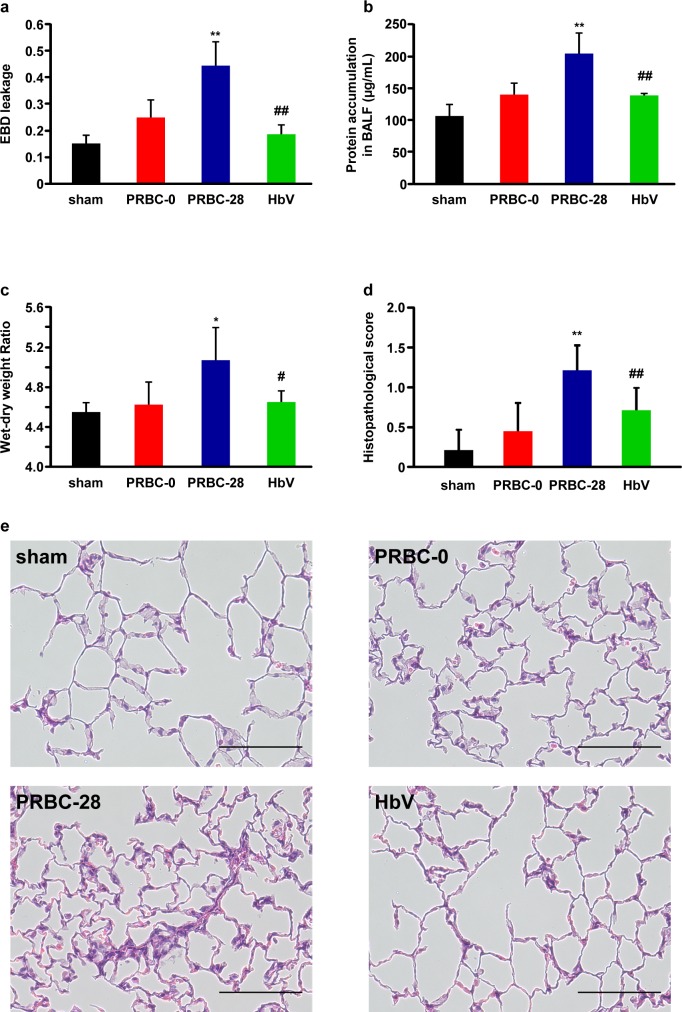
Evaluation of Pulmonary edema and lung injury after hemorrhagic shock and resuscitation with PRBC-0, PRBC-28 and HbV. (a) Percentage of Evans Blue (EBD) leakage into bronchoalveolar lavage fluid (BALF), (b) protein concentration in BALF, (c) lung wet/dry weight ratio and (d) changes in lung histopathology score at 6 hours after resuscitation with PRBC-0, PRBC-28 or HbV. Percent EBD leakage into BALF are calculated as [EBD concentration in BALF]/[EBD concentration in plasma (6 hour after transfusion)] × 100. Values are means ± SD; *n* = 5 each group. *p < 0.05, **p < 0.01 vs. PRBC-0 group, ^#^*p* < 0.05, ^##^*p* < 0.01 vs. PRBC-28 group. (e) Histopathology of lung at 6 hours after resuscitation with PRBC-0, PRBC-28 or HbV. Tissue sections were stained with HE. Scale bar = 100 μm.

The lung is known to be particularly susceptible to transfusions of one or more units of blood, and is referred to as a TRALI that develops during or within 6 hours after a transfusion [25]. Therefore, the extent of the lung injury at 6 hours after the administration of each sample was evaluated from 3 aspects: (i) EBD leakage and the protein concentration in BALF, an indication of lung infiltration; (ii) the wet/dry ratio, an indicator of the extent of lung edema; and (iii) HE staining, a histopathological indicator of the condition of the lung. As a result, EBD leakage in BALF, the protein concentration in BALF and lung wet/dry weight ratio were increased in the PRBC-28 group compared with the corresponding values for the PRBC-0 group, whereas the values for the HbV group were similar to those for the PRBC-0 group (Fig 5A–5C). Furthermore, HE staining and histopathological scores indicated that resuscitation with PRBC-0 and HbV prevented the development of lung injury, when compared to PRBC-28 resuscitation (Fig 5D and 5E). Current study reported by Stapley et al. showed that stored RBC transfusion increased mortality and induced acute lung injury in murine model of trauma-hemorrhage due to increased free heme level during storage [26]. Although we did not evaluate free heme levels in all of the resuscitative fluids used in this study, a previous study showed that HbV has a high degree of physicochemical stability with no leaking Hb from HbV after a 1 year period of storage at room temperature, suggesting that the free heme level in HbV would be negligible [16]. Therefore, the difference of free heme level in each resuscitative fluid might contribute to the extent of post-transfusion lung injury. These results indicate that resuscitation with stored HbV has less potential post-transfusion lung injury than PRBC-28 and this potential would be equivalent to that for the PRBC-0. Therefore, an HbV suspension would be not only an effective resuscitation fluid similar to fresh PRBC, as shown in Fig 2, but its use would also avoid the induction of lung injury after a transfusion, in contrast to stored PRBC.

### Inflammatory and oxidative conditions in lung tissue

**Fig 6:**
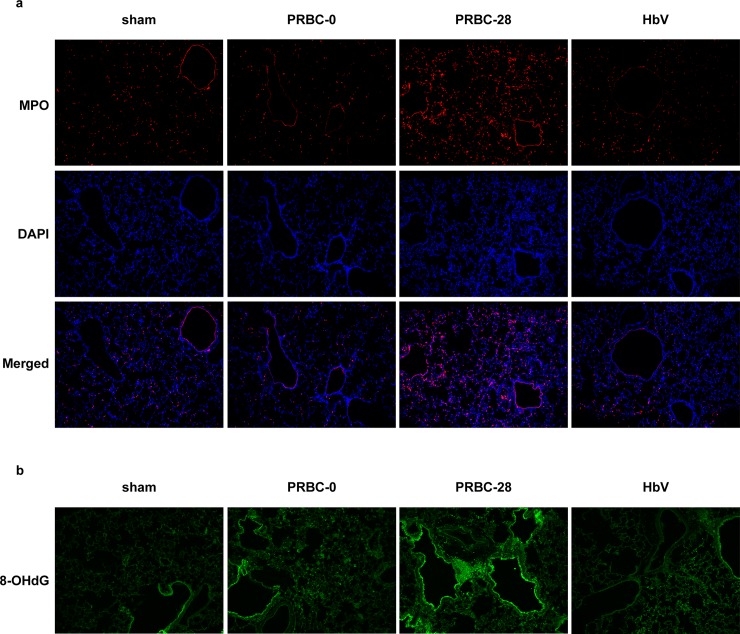
Immunological staining of lung sections. (a) Myeloperoxidase (MPO) staining and (b) 8-hydroxy-2’-deoxyguanosine (8-OH-dG) staining for evaluation of neutrophils accumulation in lung at 6 hours after resuscitation with PRBC-0, PRBC-28 or HbV. Immunofluorescent images indicated MPO (red), nuclei (blue, DAPI) and 8-OH-dG (green).

Neutrophil extravasation leads to tissue injury associated with a wide variety of factors, including free radicals. Although the mechanism responsible for the development of post-transfused lung injury is not fully understood, neutrophil activation and extravasation could, in part, be responsible for this [27]. To address this issue, we carried out immunostaining for the presence of MPO, an enzyme that is present in abundant levels in neutrophil granulocytes, to evaluate the extent of infiltration of neutrophils in lung tissue at 6 hours after the administration of each fluid. As shown Fig 6A, the accumulation of MPO was much higher in the case of the PRBC-28 preparation than for PRBC-0, while the accumulation of MPO by HbV in lung tissue was similar to that for PRBC-0. In addition, reactive oxygen species (ROS) derived from activated neutrophils are thought to be one of the main causes of tissue injury. To address this issue, we performed immunostaining of lung tissue for 8-OH-dG, an oxidation product derived from nucleic acids. Similar to the results for the immunostaining for MPO, as shown in Fig 6A, higher concentrations of 8-OH-dG accumulated in lung tissue in the case of PRBC-28 compared to PRBC-0, while the level of this oxidative stress marker for the HbV group was comparable to that for PRBC-0 (Fig 6B). It was previously reported that biologically active mediators (lipid mediators, soluble CD40L, cytokines), which accumulate over time during the routine storage of blood products, in part, cause rapid intravascular neutrophil activation, resulting in the induction of lung injury with tissue injury and the development of pulmonary edema [27,28]. Therefore, the PRBC-28 used in this study would be expected to induce lung injury *via* neutrophil activation and infiltration to lung tissue. On the other hand, HbV, prepared using high purity Hb derived from outdated RBC, would not contain such biologically active mediators and their precursors [8,29]. Furthermore, HbV stored at 4°C showed a stable dispersion state for 1 year without any observable precipitation, decomposition of vesicular components or leakage of encapsulated Hb [16]. Therefore, long-term stored HbV could be used as a resuscitating agent for the treatment of hemorrhagic shock without the induction of lung injury as well as the use of short-term stored HbV [5–7].

## Conclusion

RBC transfusions are an essential procedure for reviving patients with a massive hemorrhage. However, it is anticipated by the Red Cross Society that the supply of RBC in Japan might not be adequate in the future because the amounts of donated RBC have been decreasing gradually due to the aging of our society [30]. In addition, RBC cannot be stored in solution for long periods of time, even though the RBC shelf life approved in clinics is from 21 days to 49 days, which are different among countries [3]. These facts indicate that supplies might be limited in the future, especially in overpopulated areas and remote areas, in cases of disasters such as a great earthquake, and in an emergency situation such as a pandemic. We demonstrate herein that long-term stored HbV functions as a resuscitative fluid in hemorrhagic shock model rats and that it is comparable to fresh PRBC. Furthermore, in contrast to stored RBC, HbV suppressed post-transfusion lung injury, resulting in neutrophil accumulation and subsequent oxidative damage in the lungs, which was comparable to fresh PRBC. Therefore, HbV has the potential for use as a resuscitative fluid that can be stored for much longer periods of time than RBC and it is safe for use. In addition to functioning as a resuscitative fluid for a massive hemorrhage, the findings reported herein indicate that HbV may also be used in a variety of other applications such as placental hypoxia [31], organ storage fluids [32], pulmonary fibrosis [33], and colitis [34]. Thus, the findings reported here provide useful information to show that stored HbV has considerable promise for use as both a resuscitative fluid and in treating other related disorders. However, we did not evaluate long term survival and safety after the transfusion in this study. Moreover, the biological response after resuscitation by each fluid may be different among animal species, especially in humans. Further studies will be needed to develop a more complete understanding of these limitations in preclinical and clinical trials.
